# Improving automatic cerebral 3D-2D CTA-DSA registration

**DOI:** 10.1007/s11548-025-03412-2

**Published:** 2025-05-23

**Authors:** Charles Downs, P. Matthijs van der Sluijs, Sandra A. P. Cornelissen, Frank te Nijenhuis, Wim H. van Zwam, Vivek Gopalakrishnan, Xucong Zhang, Ruisheng Su, Theo van Walsum

**Affiliations:** 1 Department of Radiology & Nuclear Medicine, Erasmus MC, University Medical Center Rotterdam, Rotterdam, The Netherlands; 2 Department of Radiology & Nuclear Medicine, Maastricht University Medical Center, Maastricht, The Netherlands; 3 Computer Science and Artificial Intelligence Laboratory, Massachusetts Institute of Technology, Cambridge, MA, USA; 4 Intelligent Systems Department, Delft University of Technology, Delft, The Netherlands; 5 Department of Biomedical Engineering, Eindhoven University of Technology, Eindhoven, The Netherlands

**Keywords:** Cross-modality image registration, Stroke, Thrombectomy, Angiography, Deep learning

## Abstract

****Purpose**:**

Stroke remains a leading cause of morbidity and mortality worldwide, despite advances in treatment modalities. Endovascular thrombectomy (EVT), a revolutionary intervention for ischemic stroke, is limited by its reliance on 2D fluoroscopic imaging, which lacks depth and comprehensive vascular detail. We propose a novel AI-driven pipeline for 3D CTA to 2D DSA cross-modality registration, termed DeepIterReg.

****Methods**:**

The proposed pipeline integrates neural network-based initialization with iterative optimization to align pre-intervention and peri-intervention data. Our approach addresses the challenges of cross-modality alignment, particularly in scenarios involving limited shared vascular structures, by leveraging synthetic data, vein-centric anchoring, and differentiable rendering techniques.

****Results**:**

We assess the efficacy of DeepIterReg through quantitative analysis of capture ranges and registration accuracy. Results show that our method can accurately register 70% of a test set of 20 patients and can improve capture ranges when performing an initial pose estimation using a convolutional neural network.

****Conclusions**:**

DeepIterReg demonstrates promising performance for 3D-to-2D stroke intervention image registration, potentially aiding clinicians by improving spatial understanding during EVT and reducing dependence on manual adjustments.

## Introduction

Ischemic stroke, a leading cause of mortality and disability worldwide [1], presents a significant challenge to healthcare systems globally. Specifically, arterial occlusions, which disrupt blood flow to the brain, account for a vast majority of stroke cases. Endovascular thrombectomy (EVT) has become the de facto treatment for ischemic stroke caused by a large vessel occlusion [2]. This minimally invasive procedure, which involves navigating a catheter to the site of the thrombus and removing it, has greatly improved patient outcomes.

In clinical practice, 3D computed tomography angiography (CTA) is used to assess EVT eligibility, while 2D digitally subtracted angiography (DSA) provides intraoperative imaging. However, the 2D nature of DSA lacks the wealth of 3D information from CTA, hindering catheter navigation. Registering CTA to DSA could enhance therapeutic decision-making by providing interventionalists with complementary information from the CTA image [2].

Automated 3D-2D registration is typically hampered by limited capture ranges in the case of optimization-based methods or limited training data available for learning-based approaches. In this work, we develop a fully automated multistage 3D-2D registration pipeline. We first use a deep learning stage to estimate initial registrations, followed by an optimization stage that leverages differentiable rendering [3] for fast and accurate final registrations. Our method builds on traditional iterative optimization setups, such as the work by Hipwell et al. [4] for 3D-2D registration of cerebral angiograms. Furthermore, the multistaged approach builds on the work proposed by  Gopalakrishnan et al. [5], where an initial registration is performed using a convolutional neural network (CNN), and is then further refined using an iterative optimization algorithm.

## Method: DeepIterReg

**Fig. 1:**
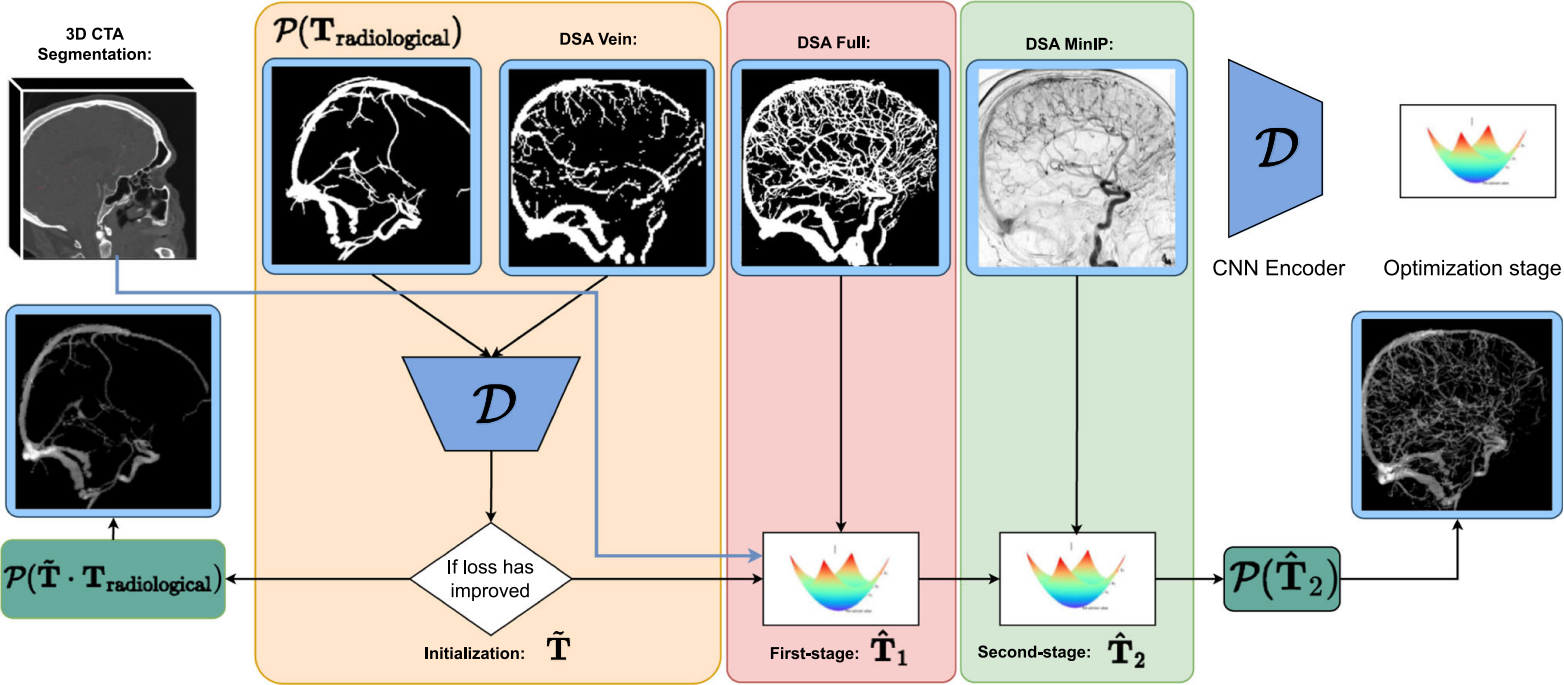
Full pipeline overview. The approach works by progressively increasing more details into the CTA with the objective of improving the registration at each step

In this section, we propose DeepIterReg, illustrated in Fig. 1, a multistage registration pipeline for 3D-2D CTA to DSA registration, combining a deep learning network with traditional optimization-based methods, powered by DiffDRR’s differentiable rendering engine [3]. We posit that in a similar approach to [5], we can perform a first-step initial registration, such that we obtain an initial pose $\tilde{{\textbf{T}}}$, using a deep neural network pre-trained on synthetic data, followed by an iterative registration approach to then compute a second, more accurate pose $\hat{{\textbf{T}}}$, where ${\hat{\textbf{T}}} \cdot {\tilde{\textbf{T}}} \approx {\textbf{T}}_{\texttt {target}}$, and ${\textbf{T}}_{\texttt {target}}$ is the target rigid transformation (registration) matrix. The initial pose is computed to overcome the limited capture range of traditional iterative-based registration techniques, where $\tilde{{\textbf{T}}}$ is the transformation that brings the CTA to its initial pose, and is *ideally* within the capture range of the iterative method employed for the later-stage registration.

### Learning-based initialization step

**Fig. 2:**
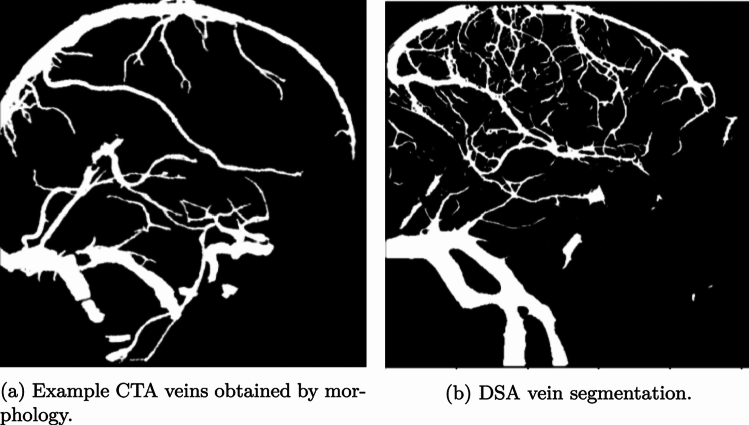
CTA and DSA veins segmentation comparison. The larger vessels typically exhibit less inter-patient variability and are present in both CTA and DSA, thereby making such structures good candidates for an initial learning-based anchoring

The initialization step aims to quickly compute an initial transformation $\tilde{{\textbf{T}}}$ that aligns the CTA to an approximate pose close to the target pose, ${\textbf{T}}_\texttt {target}$. This initial transformation serves as a starting point for further refinement in subsequent steps. We hypothesize that a convolutional neural network (CNN), trained on larger vessels that ‘surround’ the brain may be sufficient to provide an initial alignment. Visually, venous structures appear to be the most common structures in both modalities, which are typically larger and take on a semicircular structure that contours the skull. Furthermore, venous structures exhibit less inter-patient variability, making them good candidate anatomical features for a learning-based initial registration. However, such an approach would require separating the veins from the arteries in both CTA and DSA. For DSA, artery-vein separation segmentations are available thanks to the work of Su et al. [6]. For CTA, vessel segmentation is obtained using the work of Liu et al. [7]. However, artery-vein separation is not available for the CTA. In order to obtain vein isolation for the CTA, a combination of morphology and connected components was sufficient to isolate the larger veins that surround the skull. To overcome the modality difference, we binarize the resulting vein segmentation from the CTA, such that both the CTA and DSA are binarized segmentations, as illustrated in Fig. 2. These binarized vein segmentations are provided as input to the network during inference.

#### Network Architecture

**Fig. 3:**
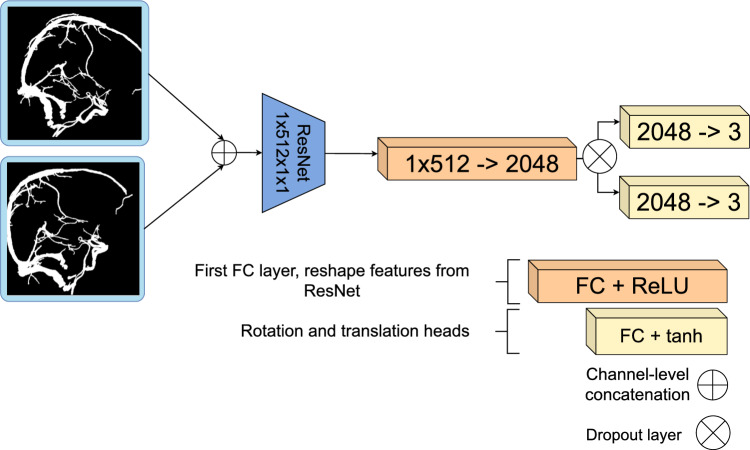
Proposed network, ${\mathcal {D}},$ extends a ResNet18 architecture to predict a rotation and translation

We use a ResNet18 backbone to extract features for the registration task, and the full architecture is given in Fig. 3.

**Fig. 4:**
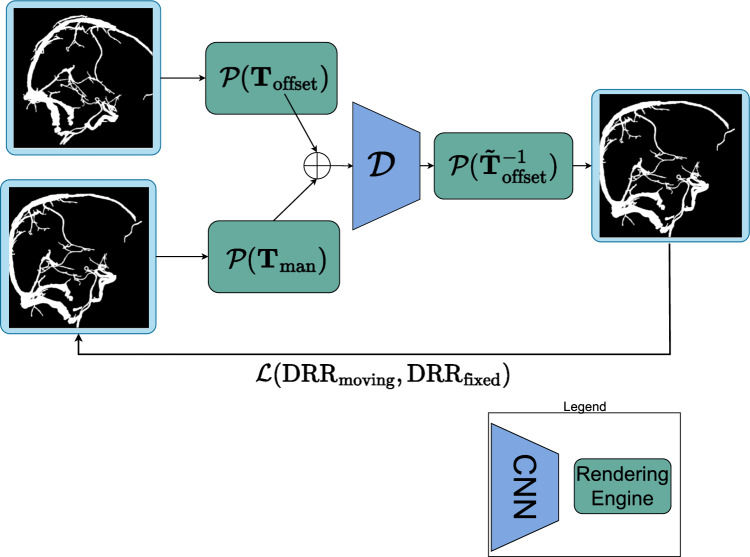
Schematic overview of the learning part of DeepIterReg. ${\mathcal {P}}$ is an instantiated drr class from DiffDRR. DRRs can be generated by supplying a pose as a parameter. The 3D CTA volume is supplied at initialization, and we therefore omit it as a parameter in Figure

We use a weighted combination of Dice loss and geodesic-based losses for rotation and translation, inspired by [5]. The total loss is defined as:

1 $$\begin{aligned} \text {Loss} =&\, \text {Dice}(\text {DRR}_{\texttt {moving}}, \text {DRR}_{\texttt {fixed}}) \nonumber \\&+ \lambda \Big ( {\mathcal {L}}_{\texttt {geo2}}({\textbf{T}}_{\texttt {pred}}, {\textbf{T}}_{\texttt {target}} ; f) \nonumber \\&+ {\mathcal {L}}_{\texttt {geo}}({\textbf{T}}_{\texttt {pred}}, {\textbf{T}}_{\texttt {target}}) \Big ) \quad , \end{aligned}$$

where ${\mathcal {L}}_{\text {geo2}}$ is the *double geodesic loss* [8], ${\mathcal {L}}_{\text {geo}}$ is the geodesic loss, and *f* is the source-to-detector distance. Both are defined on the special Euclidean group ${{\textbf{S}}}{{\textbf{E}}}(3)$, which represents rigid-body transformations consisting of a rotation and translation.

Finally, in order to perform inference on the trained model, we use real DSA-CTA pairs, the network input therefore becomes $\tilde{{\textbf{T}}} = {\mathcal {D}}({\mathcal {P}}({\textbf{T}}_{\texttt {radiological}}), \text {DSA})$, with ${\textbf{T}}_{\texttt {radiological}}$ the pose after correcting for the rotation based on the C-arm configuration from the DSA. We refer to this baseline pose as ${\textbf{T}}_{\texttt {radiological}}$.

#### Data generation and training

Due to the lack of data, we perform pre-training that relies on simulated perturbations in order to generate the training data. Specifically, we train an encoder network, ${\mathcal {D}}$, by manually perturbing a registered CTA image with a random transformation ${\textbf{T}}_{\texttt {offset}}$ applied from the registered pose. The network’s objective is to predict ${\textbf{T}}^{-1}_{\texttt {offset}}$, effectively learning how to invert the applied transformation and re-register the CTA, allowing us to generate an arbitrary amount of data and registrations. As input, we train the network using only the CTA vein segmentations: we first ‘synthesize’ a *fixed* DSA by projecting the CTA according to its registered pose. A second *moving* DRR, $\text {DRR}_{\texttt {moving}}$, is then generated by projecting the CTA according to its registered pose with the additional offset, ${\textbf{T}}_{\texttt {offset}}$. We use DiffDRR to generate the DRRs from the CTA. We call ${\mathcal {P}}(\cdot )$ the rendering engine from DiffDRR which produces the 2D DRRs, given a CTA and a transformation ${\textbf{T}}$, as input, and which is parametrized according to the C-arm configuration^1^. The final DRRs are therefore generated as follows: $\text {DRR}_{\texttt {moving}} ={\mathcal {P}}({\textbf{T}}_{\texttt {offset}})$, and, $\text {DRR}_{\texttt {fixed}} = {\mathcal {P}}({\textbf{T}}_{\texttt {man}})$.

As the original DSAs are provided as segmentations, we binarize the resulting DRRs to further emulate the downstream real CTA-DSA registration task. Examples of the CTA and DSA vein segmentations are given in Fig. 2. The venous structures contained in both segmentations are similar and represent similar anatomical features. This motivates training the network on CTA-CTA pairs, as the venous structures are sufficiently similar across both segmentations to potentially generalize to real CTA-DSA pairs. This simplifies the network’s task by eliminating non-mutual vessels and overcomes the lack of sufficient high-quality DSA images.

### Iterative refinement

Following the initialization of the CTA to an approximate target pose, the subsequent objective is to employ iterative optimization-based methods such that we achieve an accurate final pose.

**Fig. 5:**
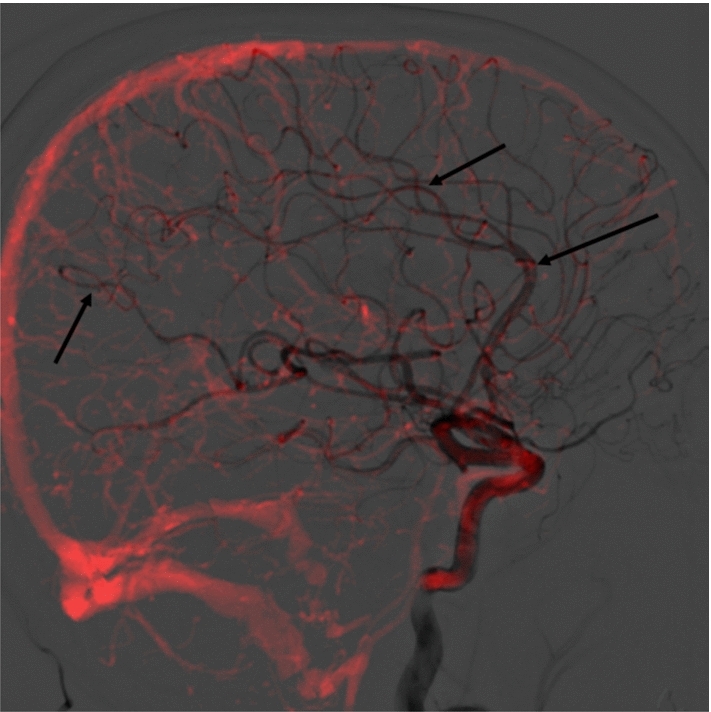
A CTA projected according to its registered pose over a DSA. CTA is shown with a red tint to enhance vessel visibility. Arrows point to locations containing smaller arteries that overlap in both the CTA and the DSA, which we hypothesize as being necessary for a finer-grained alignment

The principal challenge at this stage is to achieve an accurate final registration. As outlined previously, we hypothesized that using larger vessel segmentations is sufficient for an approximate initial pose. This is likely not the case for a highly accurate registration—crucially, smaller arteries present in both modalities are typically used when manual registration is performed. This can be illustrated visually, as seen in Figure 5, where the DSA frame is extracted from the arterial phase.

If smaller vessels are critical to an accurate registration, it will be important to make use of the full vessel tree in both modalities. To this end, we propose a two-stage iterative optimization method: an intermediary step after the initialization can be performed to further refine the initial CTA pose. The optimization algorithm requires as input a fixed reference image, in our case, the DSA, as well as the current pose of the CTA, ${\tilde{\textbf{T}}}$, obtained from the initialization. Parameter updates are performed using Adam optimizer until a predetermined number of iterations is reached, and normalized cross-correlation (NCC) as a metric to be optimized. We call the resulting transformation from the optimization stage ${\hat{\textbf{T}}}_1$.

Lastly, we posit that a further refinement of the transformation ${\hat{\textbf{T}}}_1$ can be achieved by substituting the DSA segmentation with the DSA minimum-intensity projection (MinIP) in the optimization stage defined above, while using an alternative intensity-based loss. The previous optimization approach relied on the segmentation, which effectively discards pixel intensity information from the DSA. Additionally, the optimization utilized NCC as the loss function, which measures the correlation between the overall structures of the DSA and CTA. However, a potentially more effective refinement could be achieved by substituting NCC with a distribution-based similarity measure. We define ${\hat{\textbf{T}}}_2$ as the second-stage optimization method, which runs in an identical manner to the first-stage, with smaller step sizes in the optimization step, and mutual information (MI) as a similarity metric to optimize. At both steps of the optimization procedure, we select the pose that corresponds to the highest similarity.

## Experiments and results

### Data

We use data from the MR CLEAN registry [9], a registry with data from patients who have undergone EVT for ischemic stroke from seventeen centers in the Netherlands. The dataset contains CTA scans of each patient, as well as a set of DSA images acquired both pre- and post-EVT. A segmentation algorithm was run on the CTAs to produce vein segmentations, similarly, semantic segmentation was performed on the DSA using [6], which produced a set of three segmentations for each patient: a full segmentation, a vein segmentation, and an artery segmentation.

A total of 94 lateral-view patient scans were selected from a dataset comprising AP and lateral 182 patients. The selected patients were split into 81 training patients, nine validation patients, and 20 testset patients. The dataset includes a combination of pre- and post-EVT DSAs, which were not differentiated for the purposes of this study. Patient selection for the testset images was based on the quality of the DSA: suboptimal DSA images, such as ones that suffer from motion, were removed. No selection criteria were applied to the CTA images. Manual annotations (in the form of rigid transformation matrices) were obtained by an in-house medical researcher. The registrations were performed in an in-house designed MeVisLab module.

### Metrics

The mean projection error (MPE) is used to assess the accuracy of the resulting registrations. This is computed by projecting a hypercube (eight evenly spaced points from the CTA) using both the reference standard registration matrix as well as the registration matrix from the optimization algorithm and measuring the average distance between the projected points:

2 $$\begin{aligned} \text {MPE} = \frac{1}{N} \sum _{i=1}^{N} \Vert {\textbf{p}}_i^{\text {ref}} - {\textbf{p}}_i^{\text {reg}} \Vert _2, \end{aligned}$$

where ${\textbf{p}}_i^{\text {ref}}$ are the points projected according to the reference standard, and ${\textbf{p}}_i^{\text {reg}}$ the points projected according to the obtained registration matrix.

When evaluating the capture range with a large number of perturbations (such as with the simulated poses), we construct a histogram and plot the median deviation of the points projected *before* versus the points projected *after* registration. We define the bin size such that there are 10 total bins, each containing 10% of the registrations.

### Optimization method capture ranges

To evaluate the capture ranges of the optimization method, we run a registration of the CTA to the DSA using the full dataset of 94 patients. The final accuracy of the registration is determined using MPE.

**Fig. 6:**
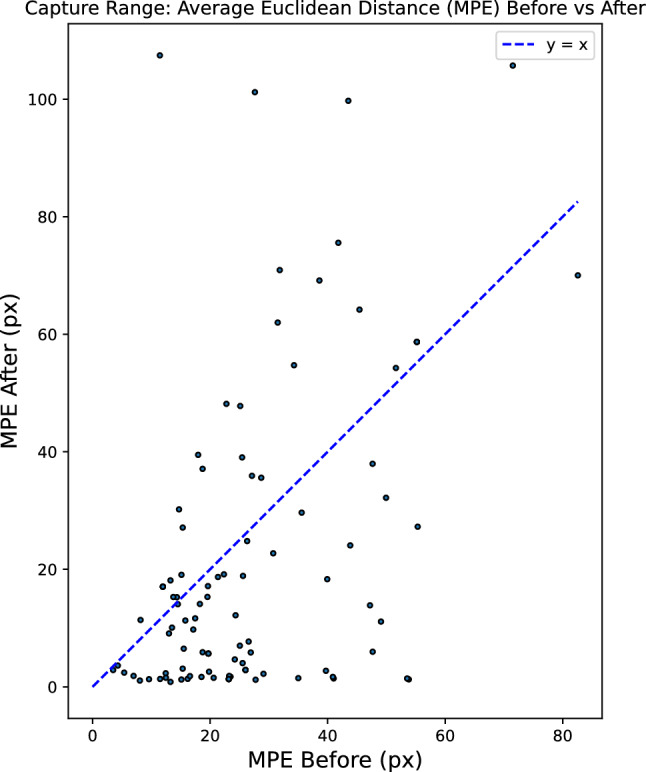
Average Euclidean distance (MPE) for all patients

Figure 6 illustrates the average Euclidean distances before and after registration when registering the 94 patients from their radiological pose. We observe an MPE *before* registration of 26 pixels, or 22.7 mm, and an MPE of 21 pixels, or 18.3 mm after registration.

**Fig. 7:**
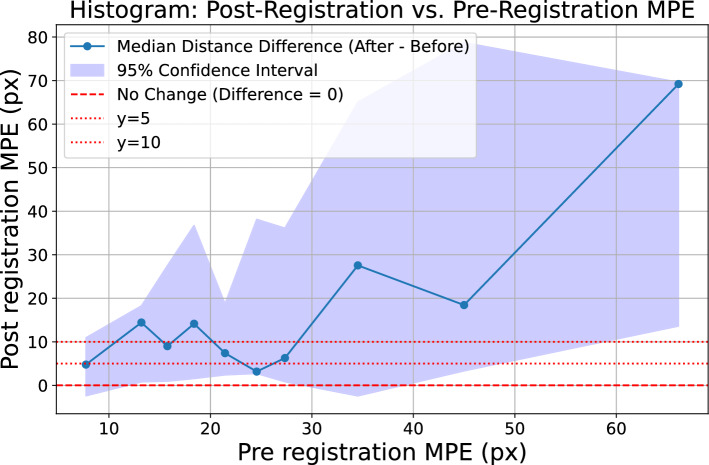
MPE before versus after registration for all 94 patients using optimization-only registration

Figure 7 contains the histogram of median deviations corresponding to Figure 6. For smaller distances, such as distances below 25 pixels, the median oscillates between 0 and 15, and eventually diverges at 25 pixels. Over all patients, taking a $y=5$ MPE threshold, we find that 37 of the 94 patients are successfully registered, corresponding to 39% of of the dataset.

### Accuracy of full pipeline

In this experiment, the full pipeline performance—and therefore the improvement from the initialization—is assessed. We perform a twofold assessment. First, using the set of 20 testset patients, we observe whether the number of successfully registered patients improves. Second, we apply perturbations to the 20 testset patients to increase the total number of registrations. This larger number of registrations can then be used to assess the median capture range improvement.

#### Testset registrations

We first evaluate the results of the 20 testset patients when registered via the optimization-only method, illustrated by the blue points in Figure 9. Using a success threshold of 5 mm, there are 11 success cases. We find the overall MPE after registration is 1.9 pixels, or 1.7 milliliters for success cases. The overall MPE over all patients is reduced from 24 pixels, or 21.1 millimeters to 11 pixels, or 9.7 millimeters.

The contribution of the initialization network is evaluated by analyzing the reduced distances in Figure 9, which highlight an improvement over the optimization-only approach. The horizontal dotted lines illustrate how the distance to the reference standard changes when the initialization is applied. All registrations below the $y=10$ except for 10153 have an optimal loss, meaning that they have a maximum theoretical loss by the end of the optimization process, thus making the total number of successful registrations 14, or 70%. The overall MPE is lowered to 8 pixels or 7.62 millimeters. For successfully registered patients, the overall MPE is 2.44 pixels or 2.13 millimeters.

#### Simulated registrations

We can further supplement these experiments by investigating the net improvement to the capture range brought by the initialization in simulated registrations.

**Fig. 8:**
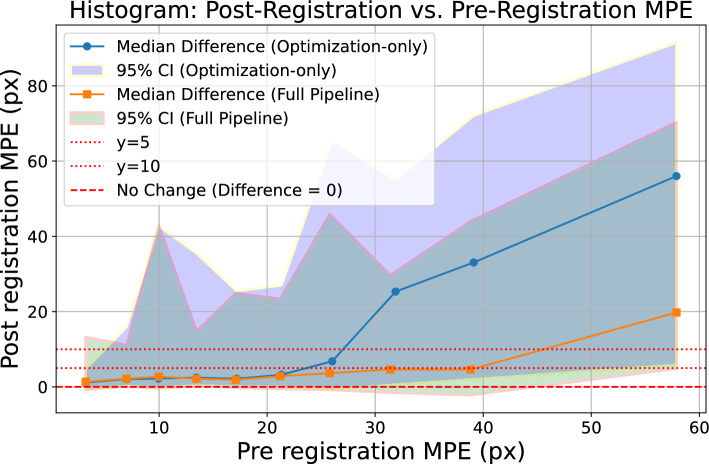
Capture range plot, optimization-only versus full pipeline

Figure 8 illustrates the change in median capture range resulting from the addition of the initialization step. Compared to the optimization-only approach, we observe that the median capture range starts to diverge from $y=5$ at 40 pixels, a net improvement over the 20 pixel median capture range identified without using an initialization.

**Fig. 9:**
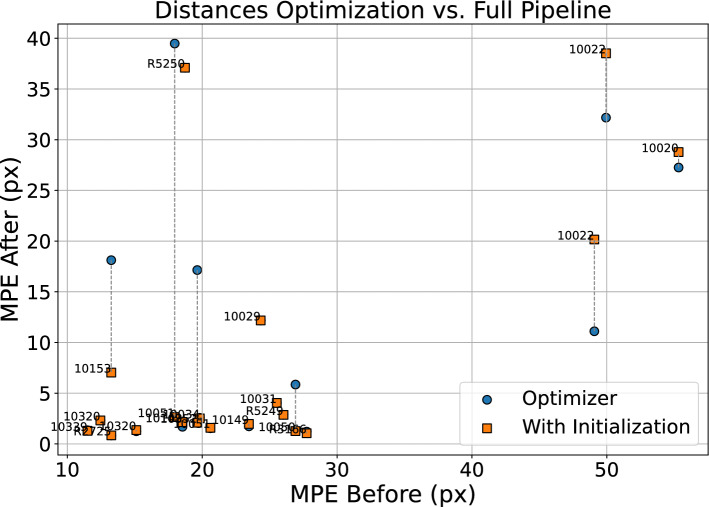
Comparison of MPE: optimizer-only versus with deep learning initialization. Dotted line shows how distance change.

### MinIP-based refinement

To assess whether an intensity-based refinement using mutual information as a loss improves the final registration result, we run the three-stage pipeline on the same set of CTA-DSA pairs as used in experiment 3.4.1.

With the addition of a third-stage intensity-based iterative refinement, we find that the MPE over all successfully registered patients decreases to 2.08 millimeters, while the number of successfully registered patients remains 14.

### Manual assessment

Finally, a visual assessment is performed by a clinical expert; this experiment aims to determine if a radiologist can reliably discriminate between registration results. This provides a qualitative assessment of the registration, and also can be used to assess the quality of the manual registrations (our reference standard) compared to the automatic registrations. To this end, we asked a neurointerventional radiologist to score the automatic registrations versus the benchmark manual reference standard registrations.

The radiologist was supplied with a blind two-way test. Our sample consists of the 14 successful registrations from Section 3.4.1. Each comparison is either between the two-stage and three-stage, the two-stage and the reference standard, or the three-stage and the reference standard, resulting in a total of 42 comparisons. The scale used is given below:

- **Scale 1**: left definitely better than right
- **Scale 2**: left is better than right
- **Scale 3**: left is the same as right
- **Scale 4**: right is better than left
- **Scale 5**: right definitely better than left

Table 1 summarizes how many times each method was preferred by the interventional radiologist. A Wilcoxon signed rank test was used to determine whether the radiologist’s rankings revealed statistically significant differences between methods. The test identified a significant difference ($p = 0.0146$) between the three-stage versus the reference standard, suggesting that the three-stage registration offers meaningful improvements over the reference standard.

**Table 1:** Preferences given to each method by an interventional radiologist

Method	Number of Times Preferred
Three-stage	13
Two-stage	9
Reference standard	6

**Fig. 10:**
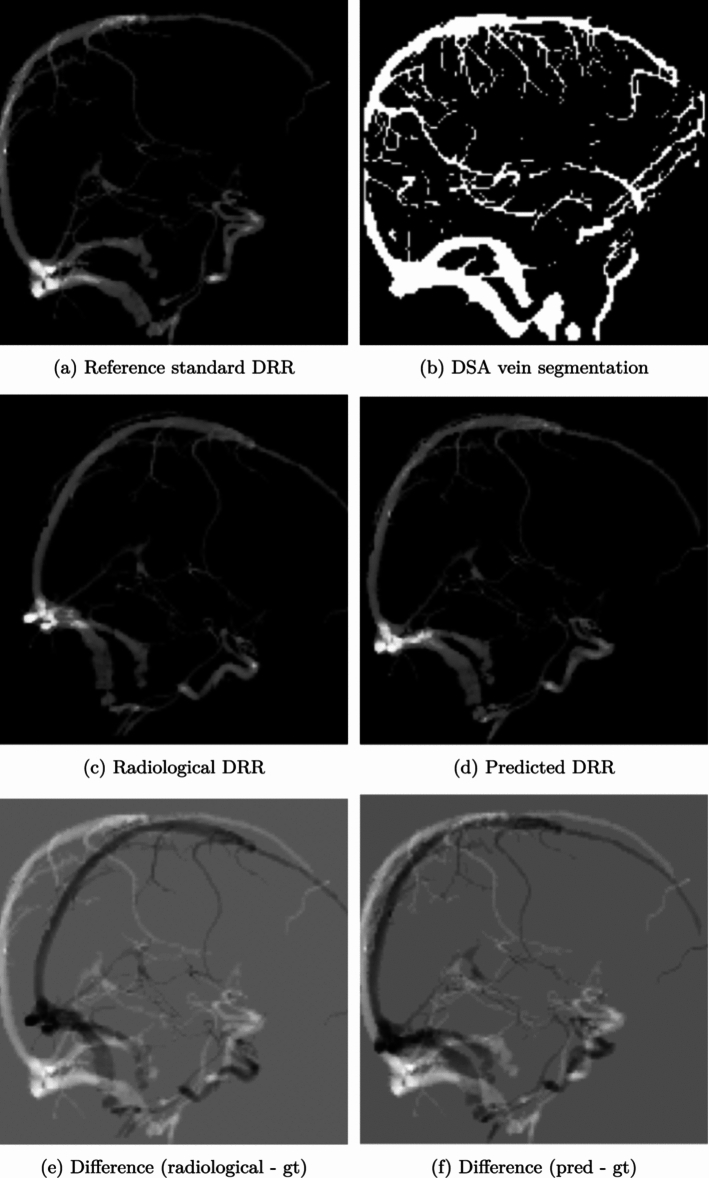
Example inputs, outputs and reference standard DRRs for the initialization network, ${\mathcal {D}}$

## Discussion

Although the method achieves a successful registration in 70% of the patients in the test set, the method is hindered by the need for high-quality DSA and CTA segmentations—correcting for sub-par CTA segmentations, we find the method accurately registers 77% of patients. Furthermore, we observe that many DSA images from the MR CLEAN dataset suffer from motion, which results in sub-par segmentations, suggesting there is still future work to be done in order to bridge the gap to the clinical viability of the proposed method. A repeat of the experiments would further benefit this study to assess the method beyond the MR CLEAN dataset.

A dataset consisting of 94 patients originating from the MR CLEAN registry were used throughout this paper. Analysis of the distances from the initial pose to the registered pose illustrates that the mean distance is 26 pixels, while the 95th percentile is 55 pixels. While the mean is within the capture range of our method, the 95th percentile is not. This limitation directly concerns the initialization stage—more extensive training is needed where the random poses used to generate DRRs are sampled from a larger range. Moreover, analysis on the distribution of each individual rotation and translation component highlights that sampling each rotation and translation component uniformly during training does not reflect an accurate distribution of offsets. We find that a patient in a CTA scanner is more likely to have a ‘slouching’ head, meaning that the offsets in the anterior-posterior direction are typically larger than those in the superior–inferior direction.

Our findings show that leveraging a pre-trained CNN, trained in a synthetic manner and on limited data, is able to generalize to predicting an initial registration pose for a real set of CTA-DSA images, when using structures mutually present in both modalities. On a test set of 20 image pairs from real EVT cases, we find the success rates of the optimization method increase from 55% to 70%.

## Conclusion

This paper introduces DeepIterReg, a multistage registration pipeline that leverages deep learning for an initial pose estimation, followed by iterative optimization to refine the registration. We find that including a deep learning network improves the median capture range from 20 to 40 pixels, and increases the number of successfully registered patents from 11 to 14, or 70%, out of a testset of 20 patients. Correcting for low-quality CTA segmentations in the testset, the method is able to successfully register 77% of the testset patients, suggesting there is a need for high-quality CTA and DSA segmentations for reliable and accurate results.

## Footnotes

1 We leave the parametrization arguments out of the function parameters for brevity. The rendering engine is initialized according to the C-arm configuration. Generating DRRs via ${\mathcal {P}}(\cdot )$ therefore also requires the CTA volume: ${\mathcal {P}}({\textbf{T}}, CTA)$. Further details are given under implementation.
